# B cell activating factor (BAFF) from neutrophils and dendritic cells is required for protective B cell responses against *Salmonella typhimurium* infection

**DOI:** 10.1371/journal.pone.0259158

**Published:** 2021-10-27

**Authors:** Runa Kuley, Kevin E. Draves, Deborah H. Fuller, Natalia V. Giltiay, Edward A. Clark, Daniela Giordano

**Affiliations:** 1 Department of Medicine, Division of Rheumatology, University of Washington, Seattle, Washington, United States of America; 2 Department of Immunology, University of Washington, Seattle, Washington, United States of America; 3 Department of Microbiology, University of Washington, Seattle, Washington, United States of America; Kurume University School of Medicine, JAPAN

## Abstract

Mice lacking B cells are more susceptible to *S*. *typhimurium* infection. How B cells contribute to protective immunity against *Salmonella* and what signals drive their activation are still unclear. Neutrophils (Nphs), monocytes (MOs), and dendritic cells (DCs) are involved in early immune responses to control the initial replication of *S*. *typhimurium*. These cells can produce B cell activating factor (BAFF) required for mature B cell survival and may help regulate B cell responses during *Salmonella* infection. Using BAFF reporter mice (BAFF-RFP^+/-^), we discovered that an i.p. infection with a virulent strain of *S*. *typhimurium* increased BAFF expression in splenic conventional DCs (cDC) and inflammatory Ly6C^hi^ MOs/DCs four days post-infection. *S*. *typhimurium* infection induced the release of BAFF from Nphs, a decrease of BAFF-RFP expression and expansion of BAFF-RFP^+^ Nphs in the spleen and peritoneal cavity. After *S*. *typhimurium* infection, serum BAFF levels and immature and mature B cell subsets and plasma cells increased substantially. Conditional knockout (cKO) mice lacking BAFF in either Nphs or cDCs compared to control *Baff*^*fl/fl*^ mice had reduced up-regulation of systemic BAFF levels and reduced expansion of mature and germinal center B cell subsets after infection. Importantly, the cKO mice lacking BAFF from either Nphs or cDCs had impaired induction of *Salmonella*-specific IgM Abs, and were more susceptible to *S*. *typhimurium* infection. Thus, Nphs and cDCs are major cellular sources of BAFF driving B cell responses, required for mounting optimal protective immunity against lethal *Salmonella* infection.

## Introduction

B cell activating factor (BAFF, also known as BLyS or Tnfsf13b) is an essential cytokine required for B-cell survival, maturation and homeostasis. It binds to three different receptors: BAFFR (BAFF receptor), TACI (Transmembrane activator and calcium-modulating cyclophilin ligand interactor) and BCMA (B-cell maturation antigen), which are predominantly expressed on mature B cells [1, 2]. Mice missing either BAFF or BAFFR lack peripheral mature B cells, underscoring the crucial function of BAFF in B cell development, response to antigens (Ags) and survival [3–5]. Increased production of BAFF has been implicated in the pathogenesis of several autoimmune diseases, including systemic lupus erythematosus, rheumatoid arthritis, and Sjögren’s syndrome [6–8]. Although less explored, BAFF also plays an important role in host responses against pathogens. BAFF is essential from protection against viral infections such as West Nile virus (WNV) and promotes WNV-specific Ab responses [9]. BAFF production in response to blood-borne T cell-independent (TI) Ags results in robust class-switched Ab responses [10–12]. BAFF also contributes to control of bacterial infections such as *B*. *burgdorferi*, *A*. *pleuropneumoniae* and *M*. *tuberculosis* by boosting humoral immune responses [13]. Increased BAFF levels due to genetic variants in the *Baff* gene results in greater protection against malaria infection [14]. Furthermore, a subset of common variable immunodeficiency patients with a mutation in the *Baffr* gene are associated with recurrent bacterial sinopulmonary infections [15], underscoring the importance of BAFF in certain bacterial infections.

We and others have shown that myeloid cells like neutrophils (Nphs), monocytes (MOs) and conventional DCs (cDCs) are major producers of BAFF during infection and inflammation [9, 12, 16, 17]. Following intestinal infection, *S*. *typhimurium* colonizes mesenteric lymph nodes and the spleen, where myeloid cells are recruited from the bone marrow (BM) to control the initial bacterial replication [18–21]. Nphs are rapidly recruited in the infected tissue upon *Salmonella* infection and actively phagocytose the bacteria to prevent their spread [19, 22]. Inflammatory MOs are also recruited to infected tissues, and in addition to being very active in taking up *Salmonella*, they are the main producers of inflammatory cytokines and inducible nitric oxide synthase (iNOS) that promotes bacterial killing [20, 21]. While DCs can produce inflammatory cytokines, they are thought to primarily play a role in Ag presentation and initiating adaptive T cell immunity against *Salmonella* [20, 23]. Myeloid cells may also provide activating and survival signals to B cells supporting adaptive immune responses [24–27]. Whether BAFF produced by myeloid cells plays a role in B cell regulation and protective immune responses to *S*. *typhimurium* has not been investigated.

Mice lacking B cells are more susceptible to virulent *Salmonella* infection, suggesting that B cells play a role during primary *S*. *typhimurium* infection [28]. While passive transfer of immune serum was protective in some studies [29, 30], another study found that the protective role of B cells during secondary infection with virulent *Salmonella* was independent of Ab secretion [31]. A number of studies suggest that B cells contribute to *Salmonella* immunity mostly during secondary infections [30–32]. However, systemic infection studies with attenuated *Salmonella* strains have shown that extrafollicular (EF) Ab responses help limit the spread of infection after primary challenge [33, 34]. How B cells contribute to immunity during an acute systemic infection of virulent *Salmonella* is less well understood. In the present study, we identified specific BAFF-producing myeloid cell populations affected by systemic *Salmonella* infection and investigated their requirement for evoking protective B cell responses. We utilized BAFF reporter mice and *Baff*^*fl/fl*^ mice we developed [9] and infected i.p. with a virulent *Salmonella* strain to investigate primary systemic immune responses to acute infection, as previous studies have done with attenuated *Salmonella* strains [18, 33]. Infection of BAFF reporter mice by *S*. *typhimurium* led to significant changes in BAFF-expressing Nph, MO, and cDC subsets. The selective removal of BAFF from Nphs or cDCs reduced the expansion of splenic B cell subsets, BAFF serum levels, and *Salmonella-*specific IgM Ab responses, revealing that BAFF produced from Nphs and cDCs is required for mounting B cell responses to *S*. *typhimurium* infection. Importantly, the cKO mice lacking BAFF from either Nphs or cDCs were more susceptible to *S*. *typhimurium* infection. Thus, BAFF produced by both Nphs and cDCs play a key role in driving protective B cell immunity against lethal *S*. *typhimurium* infection.

## Results

### Characterization of responses of BAFF-RFP^+/-^ reporter mice to *S*. *typhimurium* infection

**Fig 1:**
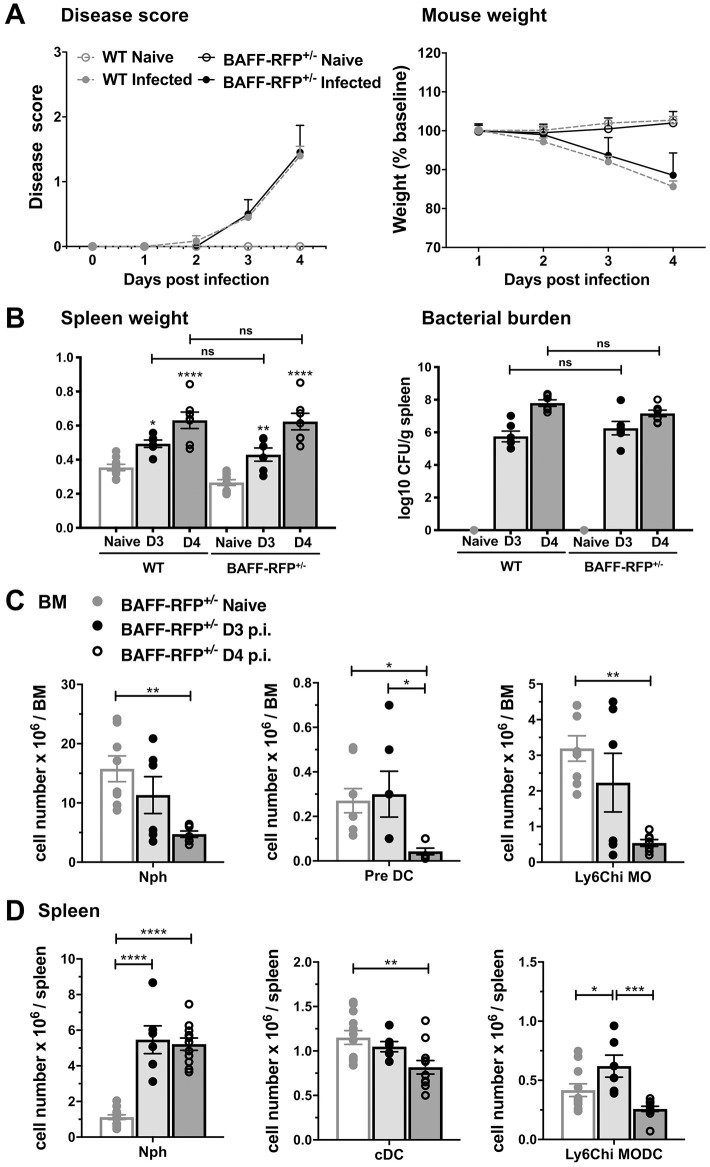
Clinical outcome and early myeloid cellular responses in BAFF-RFP^+/-^ mice after *S*. *typhimurium* infection. WT mice and BAFF-RFP^+/-^ mice were infected i.p. with 500 CFU *S*. *typhimurium*. (A) Clinical scores and mouse weights were taken daily. (B) Spleen weights and splenic bacterial burdens were measured at day 3 and 4 p.i. Data are combined from three independent experiments (n = 6–10). (C and D) Cells from BMs and spleens were harvested at day 3 and 4 p.i., and myeloid cell subsets were determined by flow cytometry. (C) Numbers of BM Nphs (CD11b^+^Ly6G^hi^Ly6C^int^SSC^int-^), Pre DCs (CD11b^+^CD11c^+^Ly6C^-^CD115^+^CX3CR1^hi^CCR2^+^ MHCII^+^Ly6G^-^SSC^-^) and Ly6C^hi^ MOs (CD11b^+^CD11c^-^CD115^+^CX3CR1^+^CCR2^hi^ MHCII^-^Ly6G^-^SSC^-^) from BAFF-RFP^+/-^ mice after *S*. *typhimurium* infection. Graphs summarize data from three independent experiments (n = 6–8). (D) Numbers of splenic Nphs (CD11b^hi^Ly6G^hi^Ly6C^int^ SSC^int-^NK1.1^-^), cDCs (CD11c^hi^CD8^+/-^B220^-^Ly6G^-^NK1.1^-^) and Ly6C^hi^ MO/DCs (CD11b^hi^ Ly6C^hi^CD11c^hi/-^SSC^-^Ly6G^-^NK1.1^-^) from BAFF-RFP^+/-^ mice after *S*. *typhimurium* infection. Data are combined from four independent experiments (n = 6–12). (A) Timeline data of clinical scores and mouse weights were analyzed by Two-Way ANOVA with Holm-Sidak’s multiple comparison test. (B-D) Bar graphs show means ± SEM; * *p* <0.05, ** *p* <0.01, *** *p* <0.001, **** *p* <0.0001, as determined by One-Way Anova with Holm-Sidak multiple comparisons test.

In order to analyze which BAFF-producing cells drive B cell activation during primary *Salmonella* infection, we used our BAFF reporter mice (BAFF-RFP), where one *Baff* allele expresses the RFP protein as a measurement of BAFF expression and the other allele expresses wild type (WT) *Baff* [9]. Although BAFF-RFP^+/-^ mice, as expected, express reduced levels of BAFF compared to WT mice, they are still capable of producing mature B cells. We first examined whether the immune responses to *S*. *typhimurium* infection of BAFF-RFP^+/-^ mice were similar to WT mice. Mice were infected i.p. with 500 CFU of virulent *S*. *typhimurium* and monitored daily for survival, clinical scores, and body weight. In agreement with previous studies, B6 mice succumbed to *S*. *typhimurium* infection within a week [18, 30, 35]. WT and BAFF-RFP^+/-^ mice showed similar signs of illness and decrease in body weights during the first 3 to 4 days post infection (p.i.) (Fig 1A), and during this period, BAFF-RFP^+/-^ infected mice showed significantly enlarged spleens and increased splenic bacterial burdens comparable to WT mice (Fig 1B).

Primary infection with virulent *S*. *typhimurium* via the oral route leads to major changes in splenic myeloid cell populations early in the course of infection [19, 21, 23]. We observed similar changes in BM and splenic myeloid cell populations in BAFF-RFP^+/-^ mice, as well as WT mice, 3–4 days after systemic *Salmonella* infection (Fig 1C and 1D and S1 Fig in S1 File). BM Nphs, cDC precursors, and inflammatory Ly6C^hi^ MOs decreased in BAFF-RFP^+/-^ mice and WT mice with disease progression (Fig 1C and S1A Fig in S1 File). In contrast, splenic Nph numbers from BAFF-RFP^+/-^ and WT infected mice substantially expanded by day 3 and remained elevated at day 4 p.i. (Fig 1D and S1B Fig in S1 File). Splenic cDCs were significantly decreased by day 4 p.i. (Fig 1D and S1B Fig in S1 File), whereas inflammatory Ly6C^hi^ MO numbers increased in the infected spleens at day 3 and later decreased as the disease progressed (Fig 1D and S1B in S1 File). As previous studies have shown, the expansion of Nphs in the spleen is most likely due to their recruitment from the BM [18–21]. Thus, BAFF-RFP^+/-^ mice showed a similar progression of disease and early recruitment of Nphs from the BM to the spleen as WT mice (Fig 1A and 1B and S1 Fig in S1 File). In addition, these data show that i.p. infection with a virulent *S*. *typhimurium* strain is a good model for examining early systemic immune responses as it leads to disease progression and changes in innate cell populations similar to those described in oral infection studies [19].

### cDC and MO subsets upregulate BAFF-RFP expression after *S*. *typhimurium* infection

**Fig 2:**
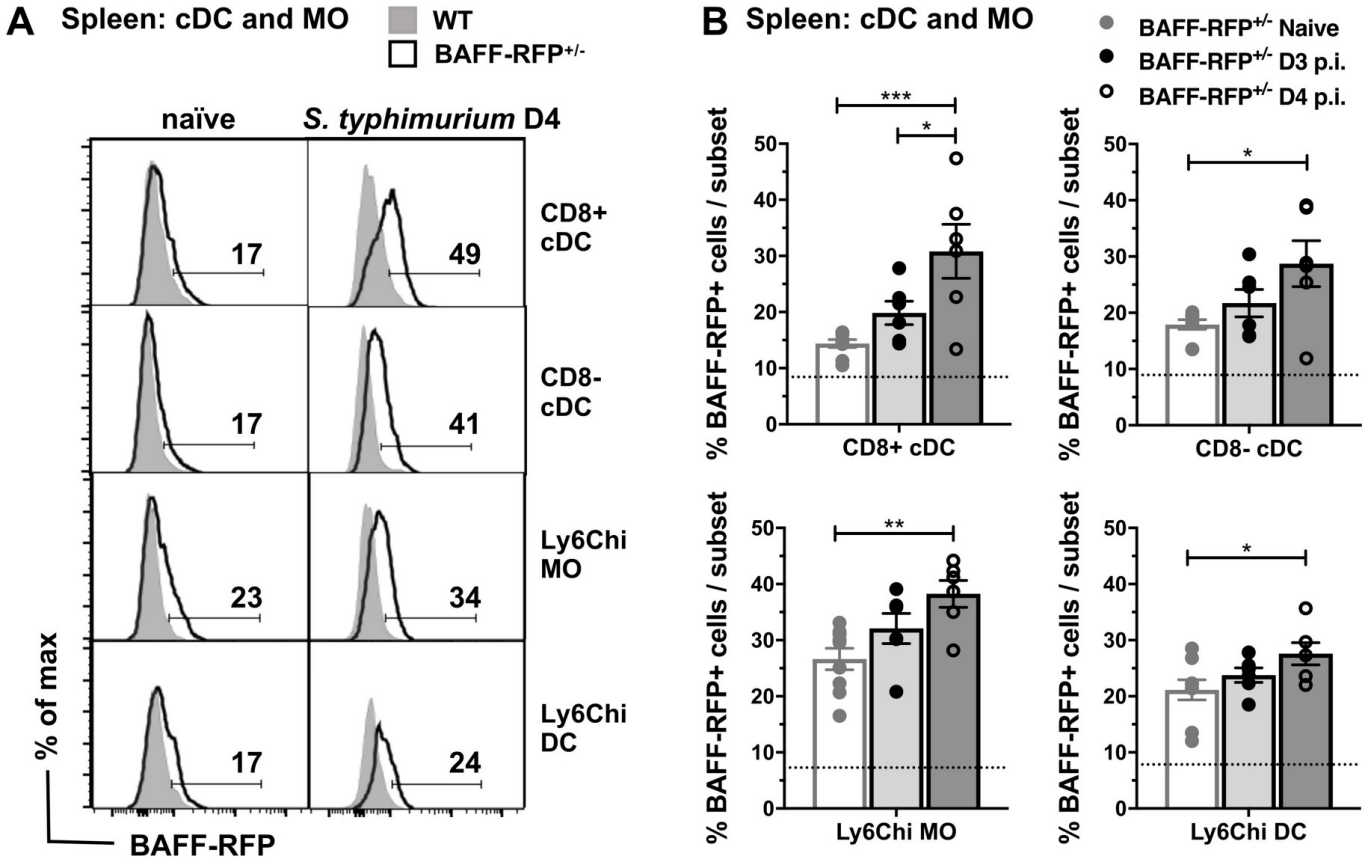
Upregulation of BAFF-RFP in cDC and MO myeloid subsets after *S*. *typhimurium* infection. WT and BAFF-RFP^+/-^ mice were infected i.p. with 500 CFU *S*. *typhimurium* and BAFF-RFP^+^ cell populations from naïve, day 3 and day 4 infected spleens were analyzed by flow cytometry. (A) BAFF-RFP expression from cDC subsets (CD8^+^ cDC and CD8^-^ cDC) and MO subsets (Ly6C^hi^ MO and Ly6C^hi^ DC) from naïve and day 4 *S*. *typhimurium* infected mice are shown as representative histograms from three independent experiments. Numbers in the plots indicate the percentage of BAFF-RFP^+^ cells. (B) Bar graphs (means ± SEM) indicate the percentage of BAFF-RFP^+^ cells and dotted lines show the percentage of RFP background signal in WT cells. Data are combined from three independent experiments (n = 6–9). Statistics were determined with one-way ANOVA with Holm–Sidak method for multiple comparisons test. * *p* <0.05, ** *p* <0.01, *** *p* <0.001.

To test whether *S*. *typhimurium* infection induced changes in specific BAFF-producing cells, we infected BAFF-RFP^+/-^ mice i.p. with 500 CFU *S*. *typhimurium* and examined BAFF-RFP expression in BM and splenic cell subsets 3–4 days p.i. In agreement with our previous report on BAFF-RFP mice and with other studies [1, 9, 36], cDCs and MOs from naïve mice expressed low levels of BAFF (Fig 2A, *left panel*). *S*. *typhimurium* infection increased the percentage of BAFF-RFP^+^ cells in splenic cDCs and MOs (Fig 2A and 2B). Both splenic CD8^+^ and CD8^-^ cDC subsets, as well as inflammatory Ly6C^hi^ MO and Ly6C^hi^ DC subsets, upregulated the BAFF-RFP signal upon infection. In contrast, BAFF-RFP expression per cell did not change in DC and MO subsets from the BMs of infected BAFF-RFP^+/-^ mice (S2A Fig in S1 File). We conclude that *S*. *typhimurium* infection upregulates BAFF expression specifically in cDCs and MOs in the spleen.

### *S*. *typhimurium* infection induces BAFF release from Nphs, expansion of splenic BAFF-RFP^+^ Nphs and increased systemic levels of BAFF

**Fig 3:**
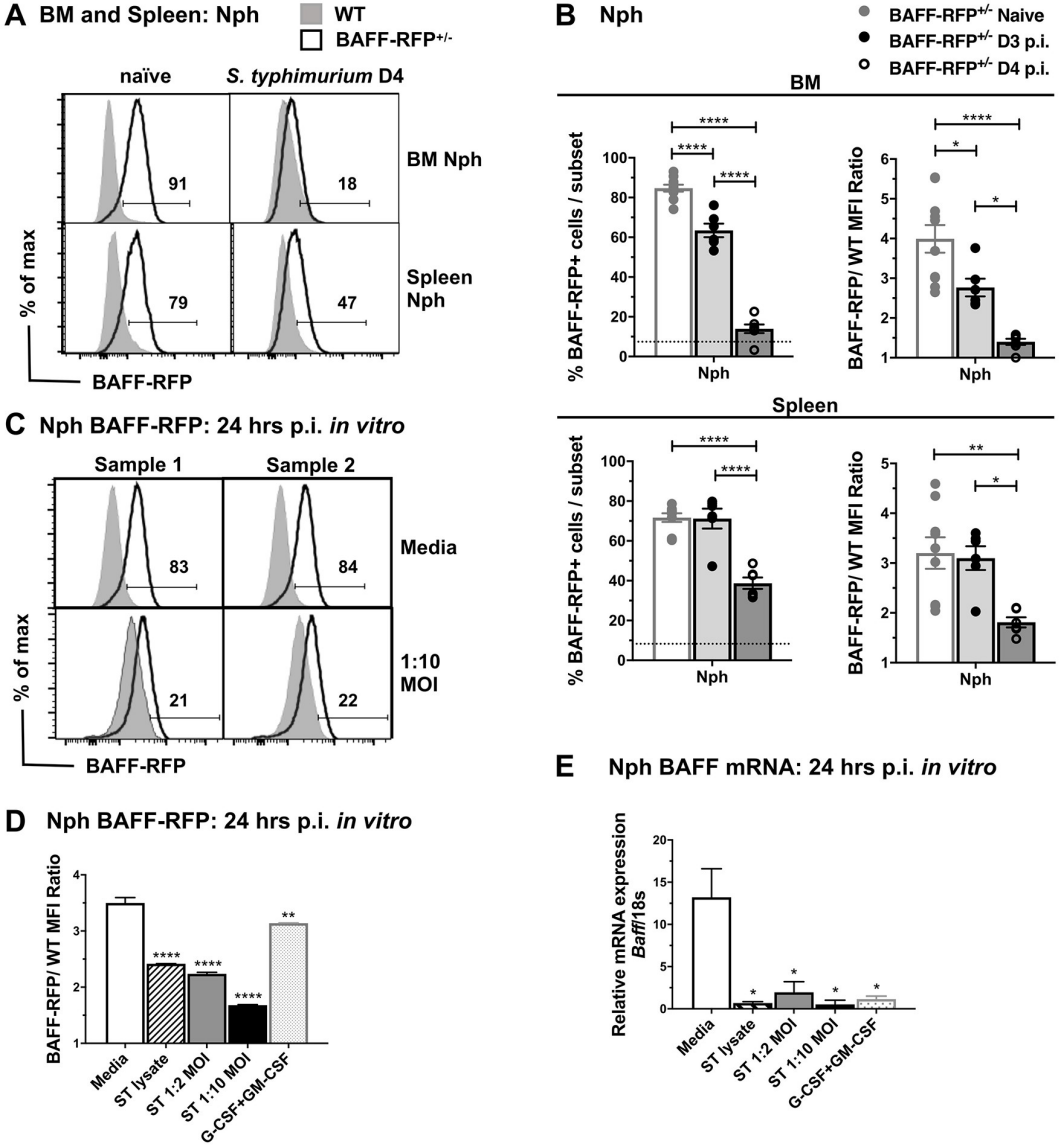
Downregulation of BAFF-RFP in Nphs after *S*. *typhimurium* infection. (A and B) WT and BAFF-RFP^+/-^ mice were infected i.p. with 500 CFU *S*. *typhimurium*, BM and splenic cell subsets were analyzed by flow cytometry. (C-E) BM Nphs were isolated from WT and BAFF-RFP^+/-^ mice and stimulated for 24 hrs with live *S*. *typhimurium* or bacterial lysates *in vitro*. (A) BAFF-RFP expression in BM and splenic Nphs are shown as representative histograms from three independent experiments. Numbers in the plots indicate the percentage of BAFF-RFP^+^ cells. (B) Graphs show the percentage of BAFF-RFP^+^ cells (*left panels*) and BAFF-RFP mean fluorescence intensity (MFI) expressed as BAFF-RFP/WT ratio (*right panels*) and summarize data from three independent experiments (n = 6–10). Dotted lines show percentage of RFP^+^ cells background in WT control. (C) BAFF-RFP expression of unstimulated BM Nphs and BM Nphs stimulated with live *S*. *typhimurium* (1:10 MOI). Data are shown as duplicate representative histograms from three independent experiments. (D) Flow cytometry data show BAFF-RFP expression as a BAFF-RFP/WT ratio of the MFI and are from one representative experiment of three independent experiments. (E) *Baff* mRNA expression analyzed by quantitative PCR and shown as arbitrary units relative to 18S. Data show one representative experiment of two independent experiments. (C-E). *In vitro* experiments with purified BM Nphs were done pooling 6–10 mice per group. In D and E, G-CSF plus GM-CSF were used as positive controls. (D and E). Bar graphs show means ± SEM; * *p* <0.05, ** *p* <0.01, **** *p* <0.0001, as determined by One-Way Anova with Holm-Sidak multiple comparisons test. (ST refers to *S*. *typhimurium*).

Nphs express the highest BAFF levels compared to other myeloid cells [9, 16, 37]. BM and splenic Nphs from naïve BAFF-RFP^+/-^ mice consistently had constitutively high levels of BAFF-RFP expression (Fig 3A) [9]. By day 3 p.i. BAFF-RFP levels in BM Nphs were downregulated and decreased further at day 4 p.i (Fig 3A and 3B, *upper panels*). A decrease in BAFF-RFP expression in splenic Nphs was only evident at day 4 p.i. (Fig 3A and 3B, *lower panels*). These data suggest that *Salmonella* directly induces activation of Nphs and the downregulation of BAFF expression. To further test this possibility, we treated purified BAFF-RFP^+/-^ BM Nphs *in vitro* with bacterial lysates or live *S*. *typhimurium*. A combination of G-CSF plus GM-CSF was used as positive control, as it is known to enhance BAFF release from Nphs [37]. 24 hrs after stimulation of Nphs with *S*. *typhimurium* the BAFF-RFP signal was decreased in Nphs (Fig 3C and 3D). As expected, since the BAFF-RFP signal is a measurement of BAFF expression [9] (see also Material and methods), we also detected significantly lower *Baff* mRNA in purified Nphs after *S*. *typhimurium* treatment compared to untreated Nphs (Fig 3E). *Baff* mRNA also decreased in WT Nphs 24 hr after stimulation with *S*. *typhimurium* (S2B Fig in S1 File). *S*. *typhimurium* downregulated *Baff* mRNA expression in WT and BAFF-RFP^+/-^ Nphs as early as 6 hrs after treatment (S2C Fig in S1 File).

**Fig 4:**
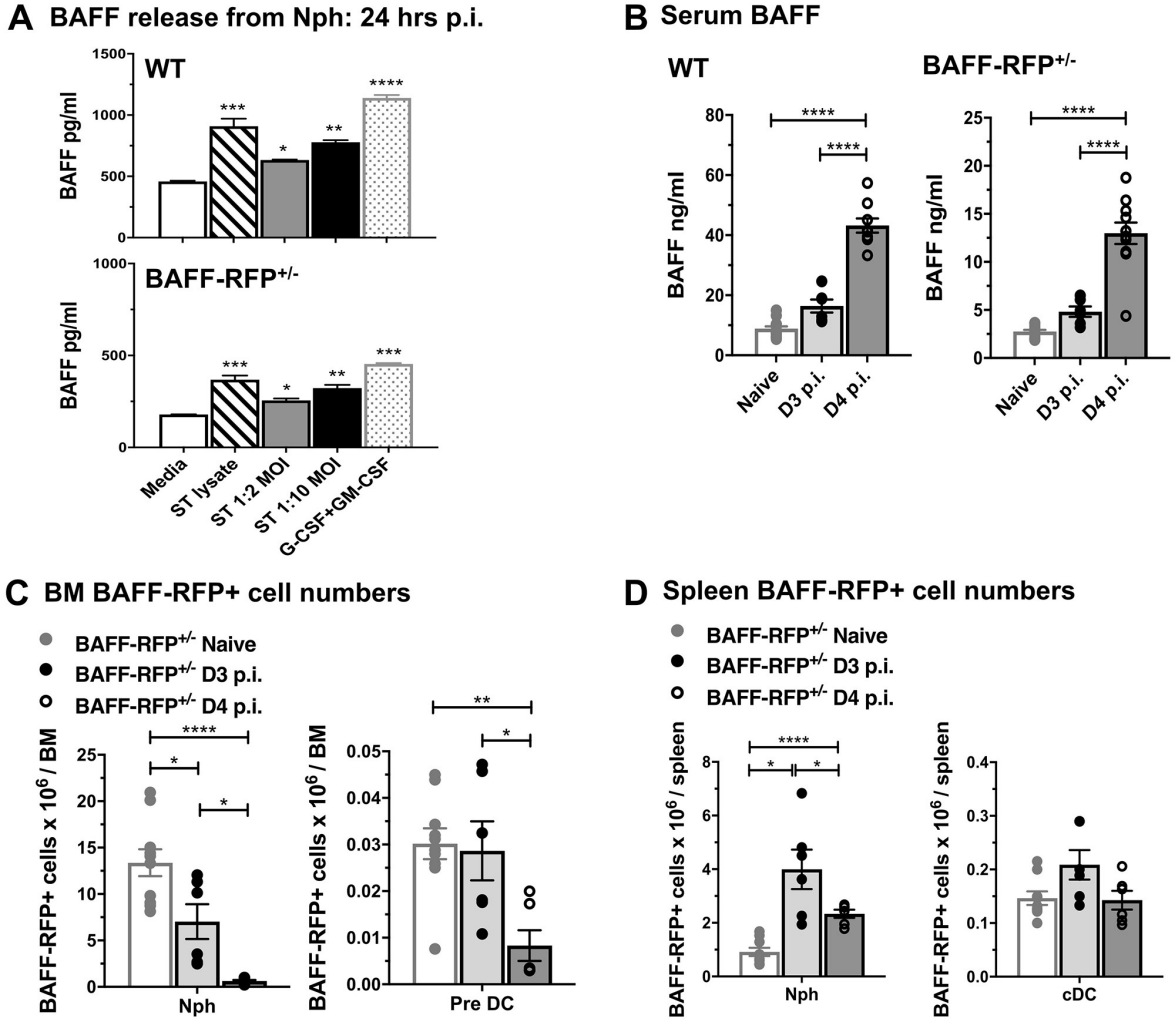
*S*. *typhimurium* induces release of BAFF from Nphs, increase in systemic BAFF levels and expansion of splenic BAFF-RFP^+^ Nphs. (A) WT and BAFF-RFP^+/-^ mice BM Nphs were isolated and BAFF was measured from supernatants of Nphs stimulated for 24 hrs with live *S*. *typhimurium* or bacterial lysates. G-CSF plus GM-CSF was used as a positive control. Data show one representative experiment of four independent experiments. (B-D) BAFF-RFP^+/-^ mice were infected i.p. with 500 CFU *S*. *typhimurium*. (B) BAFF titers in the serum of naïve and infected WT and BAFF-RFP^+/-^ mice were measured by ELISA. Graphs summarize data from four independent experiments (n = 6–14). BM (C) and spleens (D) from naïve and infected mice were analyzed by flow cytometry. Graphs show total numbers of BAFF-RFP^+^ Nphs and cDCs and summarize data from three independent experiments (n = 6–10). Bar graphs show means ± SEM; * *p* <0.05, ** *p* <0.01, *** *p* <0.001, **** *p* <0.0001, as determined by One-Way Anova with Holm-Sidak multiple comparisons test. (ST refers to *S*. *typhimurium*).

We next tested whether *Salmonella* directly induced BAFF protein release from Nphs obtained from either WT or BAFF-RFP^+/-^ mice. Stimulation with either bacterial lysates or with live *S*. *typhimurium* enhanced BAFF release in both WT and BAFF-RFP^+/-^ Nphs (Fig 4A). As expected, BAFF levels in cell supernatants from purified Nphs from BAFF-RFP^+/-^ mice were about half the levels detected in WT mice (Fig 4A). *S*. *typhimurium* triggered BAFF release from Nphs within 6 hrs (S2D Fig in S1 File). The substantial increase in BAFF release from Nphs and recruitment of Nphs from the BM to the spleen suggested that *Salmonella* might induce increased systemic levels of BAFF. Indeed, WT and BAFF-RFP^+/-^ mice infected with *S*. *typhimurium* have substantial increases in serum BAFF levels by day 4 p.i. (Fig 4B). In addition, BAFF-producing Nph numbers declined in the BM (Fig 4C) and expanded in the spleen and peritoneal cavity (Fig 4D and S2E Fig in S1 File). BAFF-RFP^+^ DC precursors decreased in the BM (Fig 4C). In contrast to Nphs, BAFF-RFP^+^ cDC did not change significantly in the spleen (Fig 4D).

We conclude that *S*. *typhimurium* infection induces: 1) the recruitment of BAFF-RFP^+^ Nphs to the spleen and peritoneal cavity; 2) BAFF release from Nphs; 3) increases in BAFF expression in splenic cDCs and MOs; and 4) at the same time, increases in systemic levels of BAFF.

### *S*. *typhimurium* infection induces the expansion of T1 B cells, FO B cells and plasma cells

**Fig 5:**
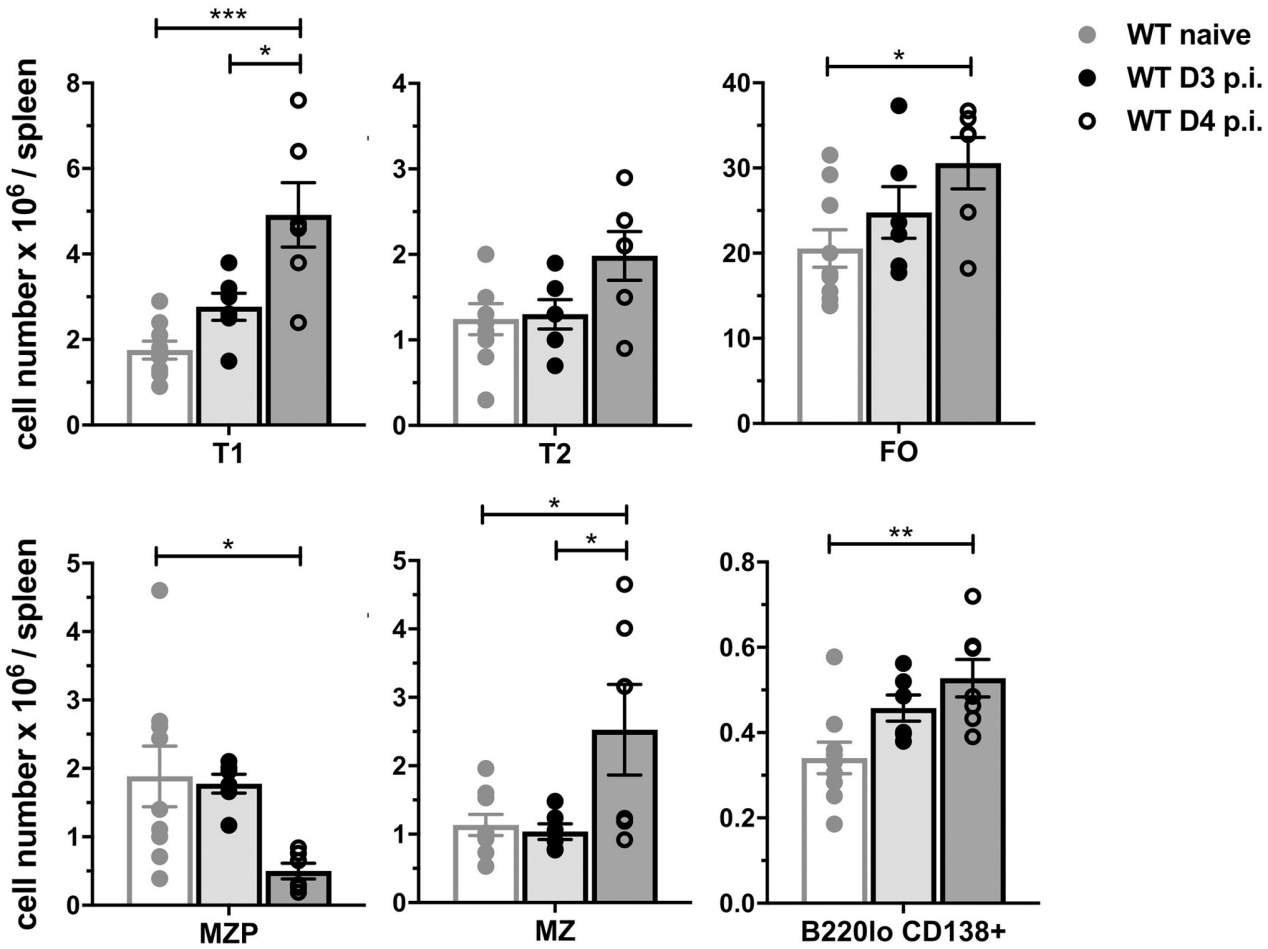
*S*. *typhimurium* induces expansion of T1, FO B cells and plasma cells. WT mice were infected i.p. with 500 CFU *S*. *typhimurium* and absolute numbers of splenic B cell subsets from naïve and infected mice were determined by flow cytometry. For gating strategy of B cell subsets see Methods and S3A Fig in S1 File. T1, T1 B cells; T2, T2 B cells; FO, follicular B cells; MZ, marginal zone, MZP, MZ precursors; B220lo CD138^+^, plasma cells. Graphs summarize data from three independent experiments (n = 6–9). Bar graphs show means ± SEM; * *p* <0.05, ** *p* <0.01, *** *p* <0.001, **** *p* <0.0001, as determined by One-Way Anova with Holm-Sidak multiple comparisons test.

As BAFF is a key B cell survival factor [1, 38, 39], we examined whether the increase in serum BAFF levels and expansion of BAFF-producing cells after *S*. *typhimurium* infection affects the expansion of splenic B cell subsets in WT and BAFF-RFP^+/-^ mice. Transitional 1 (T1) and follicular (FO) B cell numbers were significantly increased at day 4 p.i compared to uninfected WT and BAFF-RFP^+/-^ mice (Fig 5, S3A and S3B Fig in S1 File). An increase in splenic MZ B cells and a trend towards increasing T2 cells were observed in WT mice (Fig 5). In contrast, the precursors of MZ B cells (MZP) were significantly decreased in both mice strains. Moreover, splenic B220^lo^ CD138^+^ PC numbers were significantly up-regulated in WT and BAFF-RFP^+/-^ mice (Fig 5 and S3B Fig in S1 File). Thus, *S*. *typhimurium* infection, together with the expansion of myeloid BAFF-producing cells, induced changes in the splenic B cells, particularly, an expansion of T1, FO B cell and PCs.

### BAFF produced by Nphs is required for optimal B cell expansion, *Salmonella*-specific IgM responses and protection against *S*. *typhimurium* infection

We next addressed whether BAFF plays a protective role during systemic *Salmonella* infection. Previous studies showed that mice lacking B cells were more susceptible to virulent *S*. *typhimurium* primary oral infection [28]. Similarly, B cell deficient μMT mice succumbed to virulent *S*. *typhimurium* i.p. infection significantly earlier than WT mice (S3C Fig in S1 File). Therefore, B-cell mediated immunity plays a protective role during *S*. *typhimurium* infection.

**Fig 6:**
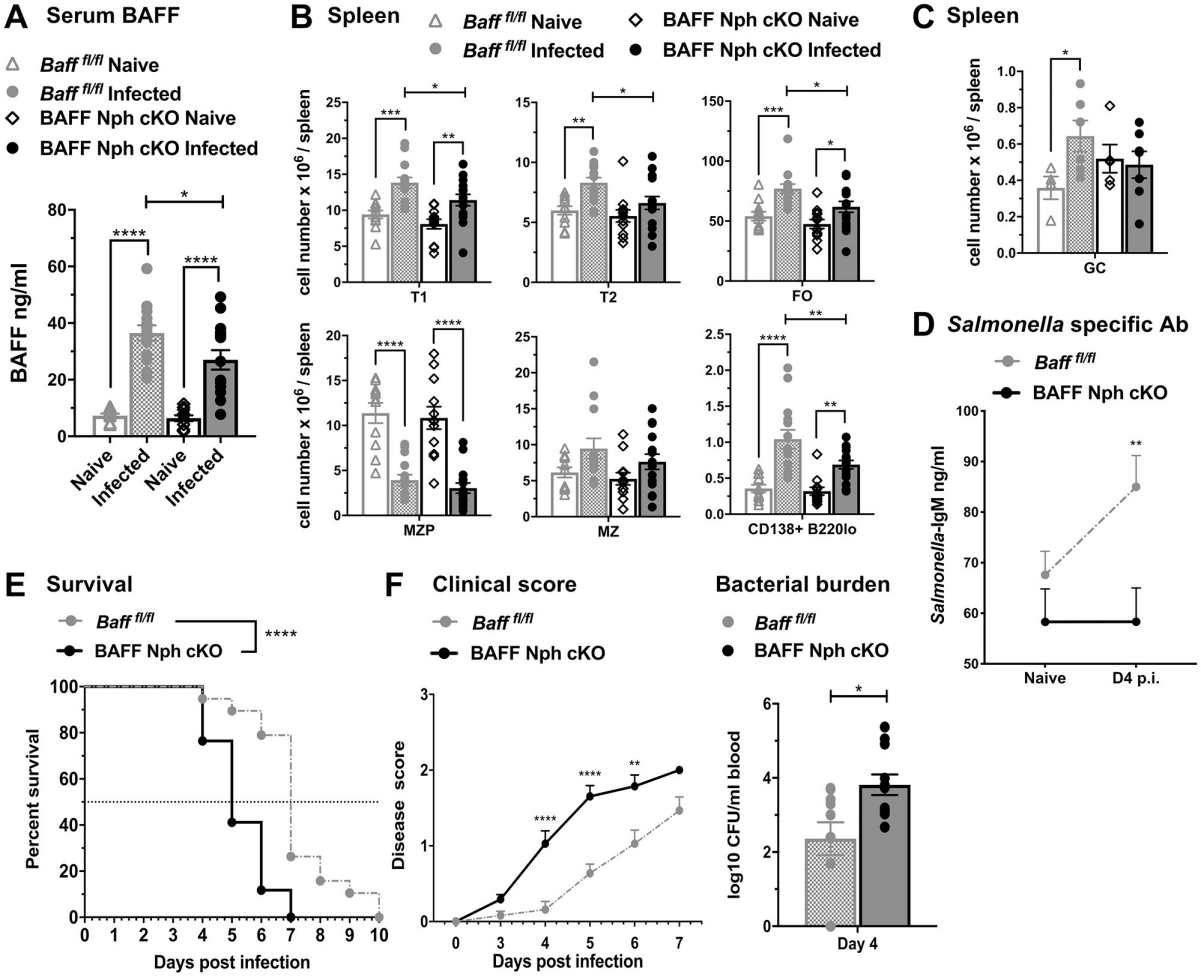
BAFF from Nphs is required for protective immune responses to *S*. *typhimurium* infection. (A-C) *Baff*^*fl/fl*^ and *Baff*^*fl/fl*^ *MRP8*^*Cre*^ (BAFF Nph cKO) mice were infected i.p. with 500 CFU *S*. *typhimurium*. Serum (A) and spleens (B and C) were harvested from naïve mice and mice infected for 4 days. (A) BAFF titers in the serum were measured by ELISA. (B and C) Absolute numbers of total splenic B cell subsets (T1, T2, FO, MZP, MZ and B220lo CD138^+^ plasma cells) (B) and GC B cells (C) were analyzed by flow cytometry. (A and B) Data are combined from four independent experiments (n = 11–15). (C) Data are combined from two independent experiments (n = 4–6). (D-F) *Baff*^*fl/fl*^ and BAFF Nph cKO mice were infected i.p. with 25 CFU *S*. *typhimurium*. (D) *Salmonella* specific IgM Abs in the serum were analyzed by ELISA. Data shown in (D) are combined from three independent experiments (n = 17–24). (E-F) Data show survival (E), clinical scores (F, *left panel*) and bacterial burdens (F, *right panel*) measured at day 4 p.i. by plating blood collected via the retro-orbital route. (E and F, *left panel*) Survival and clinical scores data are combined from three independent experiments: *Baff* ^*fl/fl*^, n = 19; BAFF Nph cKO, n = 17. (F, *right panel*) Data of bacterial burdens were summarized from two independent experiments (n = 10–11). (A-D) Statistics were determined by Two-Way Anova with Holm-Sidak multiple comparisons test. (E) Survival data were analyzed using a log-rank test for significance. (F, *left panel*) Significance of clinical scores were determined by Two-Way Anova with Holm-Sidak’s multiple comparisons test. (F, *right panel*) Unpaired Student *t* test was used to determine significance in bacterial burdens. Bar graphs show means ± SEM; * *p* <0.05, ** *p* <0.01, *** *p* <0.001, **** *p* <0.0001.

Using myeloid cell BAFF cKO mice [9], we determined which myeloid BAFF sources contribute to increases in systemic BAFF levels, expansion of splenic B cells and protective immunity after *S*. *typhimurium* infection. We first assessed the role of Nph-derived BAFF by using *Baff*^*fl/fl*^ *Mrp8*^*Cre*^ (BAFF Nph cKO) mice, where *Baff* is selectively deleted in Nphs (14). BAFF Nph cKO mice infected with *S*. *typhimurium* showed some increases in BAFF serum levels 4 days after infection, but the serum BAFF increases overall were significantly lower than in infected *Baff*^*fl/fl*^ control mice (Fig 6A). In addition, the numbers of splenic transitional T1 and T2 B cells, FO B cells and PCs were increased to a lesser extent in infected BAFF Nph cKO mice compared to control mice (Fig 6B**)**. Furthermore, while CD38^-^GL7^+^ germinal center (GC) B cell numbers were up-regulated in *Baff*^*fl/fl*^ mice, they did not expand in BAFF Nph cKO mice after *S*. *typhimurium* infection (Fig 6C). However, there was no significant difference between GC B cells from infected *Baff*^*fl/fl*^ mice and infected BAFF Nph cKO mice, suggesting that Nphs do not play a major role in GC B cell expansion. To address potential changes in B cell activation in response to *S*. *typhimurium* infection, we examined levels of CD69, an early B cell activation marker [40, 41], and MHC class II, an indicator of the Ag presenting ability of B cells [42, 43]. T1, T2 and FO B cells, but not MZ had significantly reduced up-regulation of CD69 in infected BAFF Nph cKO mice compared to *Baff*^*fl/fl*^ mice (S4A Fig in S1 File, *upper panel*s). In contrast, the changes induced by *S*. *typhimurium* infection in MHC class II expression on B cell subsets were comparable in BAFF Nph cKO mice and *Baff*^*fl/fl*^ mice (S4B Fig in S1 File, *upper panel*s). Consistent with decreased expansion of PCs, BAFF Nph cKO mice did not up-regulate *Salmonella-*specific serum IgM, that was induced in *Baff*^*fl/fl*^ mice after *S*. *typhimurium* infection (Fig 6D).

We next tested if BAFF-producing Nphs play a protective role during *S*. *typhimurium* infection. BAFF Nph cKO mice infected i.p. with 25 CFU *S*. *typhimurium* had significantly increased mortality compared to *Baff*^*fl/fl*^ control mice (Fig 6E). *Mrp8*^*Cre*^ mice were used as additional controls and had similar survival rate and systemic bacterial burdens as *Baff*^*fl/fl*^ mice (S5A Fig in S1 File). The infected BAFF Nph cKO mice also showed more advanced signs of illness and higher systemic bacterial titers than *Baff*^*fl/fl*^ mice (Fig 6F). We conclude that during *Salmonella* infection BAFF produced by Nphs contributes to the increased systemic BAFF levels, expansion and activation of splenic B cells and PCs, *Salmonella*-specific IgM responses. and plays a protective role against *S*. *typhimurium* infection.

### BAFF produced by cDCs is required for B cell expansion, *Salmonella*-specific IgM responses and protection against *S*. *typhimurium* infection

**Fig 7:**
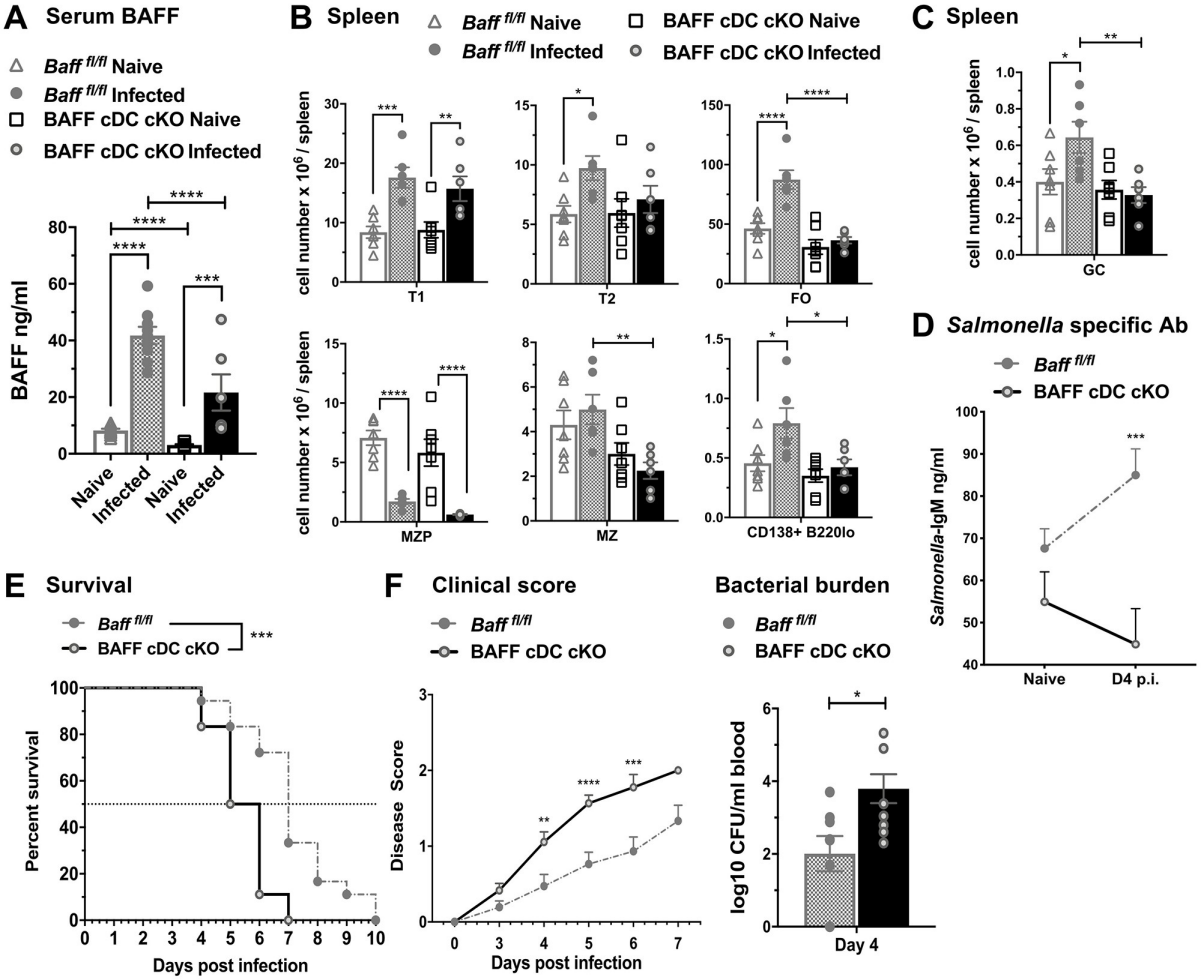
BAFF from cDCs is required for protective immune responses to *S*. *typhimurium* infection. (A-C) *Baff*^*fl/fl*^ and *Baff*^*fl/fl*^ *zDC*^*Cre*^ (BAFF cDC cKO) mice were infected i.p. with 500 CFU *S*. *typhimurium*. Serum (A) and spleens (B and C) were harvested from naïve and day 4 infected mice. (A) BAFF titers in the serum were measured by ELISA. (B) Absolute numbers of total splenic B cell subsets (T1, T2, FO, MZP, MZ and B220lo CD138^+^ plasma cells) and (C) GC B cells were determined by flow cytometry. (A-C) Data are combined from two independent experiments (n = 6–14). (D-F) *Baff*^*fl/fl*^ and BAFF cDC cKO mice were infected i.p. with 25 CFU *S*. *typhimurium*. (D) *Salmonella* specific IgM Abs in the serum were measured by ELISA. (D) Data are combined from three independent experiments (n = 17–19). (E and F) Data show survival (E), clinical scores (F, *left panel*) and bacterial burdens (F, *right panel*) were measured at day 4 p.i. by plating blood collected via the retro-orbital route. (E and F, *right panel*)) For survival studies and clinical scores, data were combined from two independent experiments: *Baff* ^*fl/fl*^ and BAFF cDC cKO n = 18. (F) Data of bacterial burdens were summarized from two independent experiments (n = 8–9). (A-D) Statistics were determined by Two-Way Anova with Holm-Sidak multiple comparisons test. (E) A log-rank test for significance was performed to analyze survival data. (F, *left panel*) Significance in clinical scores were determined by Two-Way Anova with Holm-Sidak’s multiple comparisons test. (F, *right panel*) unpaired Student *t* test was used to determine significance in bacterial burdens. Bar graphs show means ± SEM; * *p* <0.05, ** *p* <0.01, *** *p* <0.001, **** *p* <0.0001.

DCs play a significant role in the activation of adaptive immunity during the course of *Salmonella* infection [20, 23]. Splenic cDCs up-regulate BAFF expression during systemic *S*. *typhimurium* infection (Fig 2). Thus, we assessed if BAFF production by cDCs plays a role in B cell responses and protective immunity after infection. Uninfected *Baff*^*fl/fl*^ *zDC*^*Cre*^ (BAFF cDC cKO) mice [9], where BAFF is selectively depleted in cDCs, showed lower serum BAFF levels compared to controls (Fig 7A). These data indicate that in contrast to BAFF from Nphs, BAFF from cDC contributes to the homeostasis of systemic BAFF. After *S*. *typhimurium* infection BAFF cDC cKO mice increased serum BAFF levels to a lesser extent than *Baff*^*fl/fl*^ control mice (Fig 7A). However, in contrast to Nph BAFF-depleted mice, the lack of BAFF from cDCs completely prevented the increases in FO B cells and PC numbers observed in control mice (Fig 7B). Also, after infection, MZ B cells were significantly lower in BAFF cDC cKO mice compared to *Baff*^*fl/fl*^ mice (Fig 7B). Moreover, the expansion of splenic GC B cells observed in *Baff*^*fl/fl*^ mice after *S*. *typhimurium* infection, was not detected in the BAFF cDC cKO mice (Fig 7C).

Interestingly, in contrast to BAFF depletion in Nphs, BAFF depletion in cDCs did not affect CD69 up-regulation triggered by *S*. *typhimurium* infection (S4A Fig in S1 File, *lower panels*). However, the MHC II up-regulation occurring in MZ and MZP B cells from *Baff*^*fl/fl*^ control mice upon *S*. *typhimurium* infection was completely ablated in BAFF cDC cKO mice (S4B Fig in S1 File, *lower panels*). As in the BAFF Nph cKO mice, the increase in *Salmonella-*specific serum IgM Ab that occurred in control mice after infection, did not occur in BAFF cDC cKO mice (Fig 7D). Finally, similar to the BAFF Nph cKO mice, BAFF cDC cKO mice also had increased mortality (Fig 7E), higher clinical scores and bacterial burden (Fig 7F) compared to the *Baff*^*fl/fl*^ control mice, suggesting that BAFF from cDCs also plays an important protective role in *S*. *typhimurium* infection. Survival rates and systemic bacterial burdens were not significantly different in control *zDC*^*Cre*^ mice and *Baff*^*fl/fl*^ mice (S5B Fig in S1 File). In contrast to BAFF Nph cKO mice and BAFF cDC cKO mice, the mortality of BAFF MO cKO mice was comparable to *Baff*^*fl/fl*^ control mice (S5C Fig in S1 File), suggesting that BAFF from MOs does not contribute to protection against *Salmonella* infection.

We conclude, that BAFF produced by both Nphs and cDCs is required for optimal specific humoral responses and protective immunity against *Salmonella* infection. Interestingly, BAFF from Nphs and cDCs regulate B cell responses differently. Nph-derived BAFF, but not BAFF from cDCs, regulate immature B cells. In contrast, unlike Nph-derived BAFF, cDC-derived BAFF is indispensable for mature B cell expansion in response to *Salmonella* infection.

## Discussion

Innate immune cells such as Nphs, cDCs and MOs contribute to immunity against a number of infections, including *Salmonella* [20, 44–47]. The role of innate immune cells goes far beyond pathogen killing and involves production of cytokines that critically link the innate and adaptive immune system [13, 24–26]. B cells are major contributors to protective acquired immunity to *Salmonella* infection [48]. Here we showed that BAFF produced from two different innate myeloid cell sources—Nphs and DCs—regulate B cell responses and are required for optimal protective immunity against *S*. *typhimurium* infection.

To our knowledge Nph-derived BAFF has not been previously shown to contribute to protective immune responses against infectious diseases. In our previous study, BAFF from Nphs was not required for protective immunity to WNV [9]. BAFF secretion from CD11b^+^ cells in response to the parasite *Trypanosoma cruzi* was reported to contribute to protection by expansion of total mature B cells [49]; however, which CD11b^+^ cell subsets were the essential sources of BAFF during *T*. *cruzi* infection was not investigated. Deniset et al. reported that splenic Nphs interact with B cells in the red pulp and marginal zones, promote T cell-independent (TI) Ab responses and contribute to systemic clearance of *Streptococcus pneumoniae* [50]. However, whether Nphs helped B cell responses to *S*. *pneumoniae* via BAFF was not addressed. Here we definitively demonstrated that Nphs-derived BAFF plays a protective role during *Salmonella* infection. In addition, we demonstrated that Nph-derived BAFF significantly contributes to increased systemic BAFF levels, is required for the induction of *Salmonella*-specific IgM responses, and plays a major role in sustaining B cell expansion during *S*. *typhimurium* infection (Fig 6). In agreement with our results, Coquery et al. found that in an autoimmune hyper inflammatory environment, activated Nphs may contribute to excessive serum BAFF levels and promote B cell responses [51]. BAFF produced by Nphs is required not only for *Salmonella*-driven expansion of mature B cells and PCs, but also for the optimal expansion of newly formed/transitional B cell (NF-B cell) subsets (T1 and T2 B cells). These data are particularly interesting, given the co-localization of NF-B cells and Nphs in the splenic red pulp [52]. A growing number of studies support a role for NF-B cells in protection against infections [52].

During infection, when splenic Nphs are exposed to microbial and inflammatory signals, they acquire “B helper” functions and work together with B cells to promote adaptive immune responses [27, 53, 54]. In addition to the red pulp, Nphs colonize the peri-MZ areas of the spleen where, via BAFF and other factors, they help generate TI antimicrobial Ab responses by promoting MZ B cell differentiation into PCs [24, 55]. Nph B cell helpers via production of Pentraxin 3, a complement activator, activate MZ B cells and elicit class switching and plasmablast expansion, promoting TI and T-cell dependent responses to *S*. *pneumoniae* infection [56]. Whether Nphs directly interact with mature B cells like FO and GC B cell remains controversial [54]. Recent studies have implicated recruitment of Nphs in splenic B-cell follicles and GCs in the presence of appropriate inflammatory signals [24]. Upon *Salmonella* infection, optimal expansion of NF-B cells, PCs as well as mature B cell subsets are reduced in mice lacking BAFF in Nphs. An attractive model is that during *Salmonella* infection, some Nph-BAFF in the EF area and peri-MZ zone support NF-B cells and PCs expansion, while other Nphs infiltrate the B cell follicle and via BAFF support the expansion of FO B cells. Together our data highlights BAFF-production and regulation of B cells as new important Nph function essential for host survival to *Salmonella* infection [20].

In addition to changes in BAFF-producing Nphs, splenic cDCs and inflammatory MOs upregulate BAFF expression upon *Salmonella* infection. The lack of BAFF from MOs did not affect mouse survival, suggesting that MO-derived BAFF does not play a role in protective immunity to *Salmonella*. However, mice lacking BAFF in cDCs were more susceptible than control mice to *S*. *typhimurium* infection (Fig 7). We have previously shown that cDC-BAFF, in contrast to Nph-BAFF, plays an important role in protective B cell responses against viral infection [9]. Another study suggested DCs by upregulating BAFF expression contribute to protective humoral immune responses against *A*. *pleuropneumoniae* infection [57]. Interestingly, BAFF from cDCs plays a more prominent role than Nph-BAFF in contributing to systemic BAFF levels and B cell responses against *Salmonella* infection: whilst FO B cell and PC expansion was reduced in BAFF Nph cKO mice, it was completely abrogated in BAFF cDC cKO infected mice. Previous studies have suggested that BAFF produced by DCs may drive PC responses [12, 58]. LPS from Peyer’s patch resident commensal bacterium *Alcaligenes* enhanced BAFF production by DCs, promoting antigen-specific IgA production [59]. Our results demonstrate that DC-BAFF is required for PC expansion *in vivo* during bacterial infection. In contrast to Nphs, DCs are key players in adaptive immunity to *Salmonella* [20, 60, 61]. cDCs are required to activate *Salmonella*-specific T cells and during acute *Salmonella* infection cDCs subsets are rapidly activated and reorganized within the spleen [20, 23]. Upon *S*. *typhimurium* infection a subset of CD8^-^ cDCs is redistributed from the MZ to the B cell follicles, while other CD8^-^ cDCs and CD8^+^ cDCs are increased in the red pulp, in addition to their normal presence in the T-cell areas [23]. As cDC-BAFF is required for FO and GC B cells expansion, it is possible that cDC redistribution in the B cell areas is key to support FO and GC B cell responses. Further studies are needed to address cross talk and localization of Nph and cDC with B cells in the spleen during *Salmonella* infection.

Although BAFF from both Nphs and cDCs plays a major role in regulating B cells, these BAFF sources affect B cell responses to *Salmonella* infection differently. First, Nph-BAFF regulates T1 B cells while DC-BAFF does not. Second, BAFF from Nphs, but not from cDCs, contributes to *Salmonella-*induced CD69 up-regulation in several B cell subsets. Third, BAFF from cDCs, but not Nphs, is required for MHC class II activation in MZ and MZP B cells. Either location in the spleen or specific crosstalk with B cells may explain the diverse effects on B cell activation by Nph-derived or cDC-derived BAFF. Our results suggest that while Nph-BAFF regulates early B cell activation [41], cDC-BAFF may affect the Ag presenting ability of B cells (33,76,77). MZ and MZP B have the highest MHC class II expression among B cells [43, 53]. In addition, the location of cDCs in the splenic MZ makes them good candidates to support MZ B cell Ag presenting capability via BAFF [62, 63]. Earlier studies have suggested that during primary infection, B cell Ag presentation, but not Ab secretion, may help the development of protective T cell immunity against secondary *Salmonella* infection [31, 32, 64].

Infection with attenuated *Salmonella* leads to IgG production during EF responses and a delayed GC response [33, 34]. In contrast, primary infection with virulent *Salmonella* induced an unexpected early expansion of GC B cells that required BAFF produced by cDCs. At these early timepoints after infection we did not detect any change in *Salmonella*-specific serum IgG and IgA Abs in either control *Baff*^*fl/fl*^ mice, BAFF cDC cKO mice or BAFF Nph cKO mice. However, the surprising early increase in GC B cells suggests that systemic primary infection with virulent *Salmonella* may induce a rapid adaptive immune response. Consistent with this possibility, McSorley et al. reported that *Salmonella*-specific T cell responses in mesenteric lymph nodes and Peyer’s patches occur within hours after oral infection with virulent *Salmonella* [65]. Since systemic *Salmonella* infection rapidly targets the spleen, it is possible that *Salmonella*-specific T cells develop at early time points in this model. Further studies are needed to determine whether after acute systemic infection, BAFF from splenic cDCs or Nphs regulates the ability of B cells to present Ags, induces localized changes in class-switched Abs and/or activate protective *Salmonella*-specific CD4^+^ T cells [48].

BAFF from Nphs and cDCs is also required for *Salmonella*-specific IgM production after infection. *Salmonella*-specific IgM Abs produced early after infection opsonize extracellular bacteria controlling tissue colonization and rapid increase in systemic bacterial burdens [33]. The fact that mice lacking BAFF in either Nphs or cDCs have higher bacterial levels in blood than control mice, thus, could be due to a deficiency in specific IgM Abs. Our findings are consistent with previous studies showing that Abs are required for vaccines to protect against infection with virulent *Salmonella* [30, 66]. B1 B cells rapidly produce IgM and protective Ab responses against *S*. *typhimurium* [67]. Our data do not rule out a role for other B cell subsets like B1 B cells in contributing locally to IgM production.

A limitation of our study is that infected mice die within a week after systemic infection with virulent *Salmonella*. However, our results clearly indicate that: 1) BAFF plays a protective role during primary *Salmonella* infection, and 2) this is most likely due to the regulation of B cells, given that BAFF-producing DCs and Nphs are required for the early up-regulation of specific B cell subsets and the *Salmonella*-specific IgM Abs induced by systemic acute infection. Further studies are needed to determine whether BAFF and B cell protective role occurs via IgM response, antigen presentation or cytokine production.

Stimulation with bacterial Ags induces BAFF release from DCs, MO and macrophages *in vitro* [13, 57]. Here we report that *Salmonella* directly and rapidly induces BAFF release from Nphs. Nphs constitutively express high levels of BAFF during homeostatic conditions [9, 37]. Nphs are the main infiltrating cell population during acute inflammatory responses to *Salmonella* [22, 68], and as we report here, are the predominant BAFF source expanded in the spleen and peritoneal cavity after systemic *Salmonella* infection. Cytokines produced by innate cells in response to inflammation can activate Nphs and induce BAFF release [37, 69, 70]. Virulent *Salmonella* induced BAFF release from Nphs *in vitro* to the same extent as a combination of G-CSF and GM-CSF, a potent inducer of BAFF release from Nphs [37]. Interestingly, *S*. *typhimurium* while inducing BAFF release from Nphs, also downregulated BAFF expression *in vivo* and *in vitro*. TNFα treatment also induced BAFF secretion and down-regulation of BAFF expression in human Nphs, similar to what we report here in mouse Nphs treated with *S*. *typhimurium* [71]. BAFF secretion and down-regulation of BAFF expression were induced *in vitro* by *Salmonella* within 6 hrs, timing comparable to cytokine-induced BAFF release from mouse Nphs [37]. These effects were not only rapid, but also driven by a *Salmonella* lysate as well as by live bacteria, suggesting that either bacterial -PAMPS or -proteins can directly affect BAFF in Nphs. However, it is also possible that inflammatory cytokines rapidly induced by *S*. *typhimurium* infection play a role [72].

Our study highlights the importance of the relationship between innate cells and adaptive B cell responses during *Salmonella* infection. Although earlier studies have implicated B cells in protection during secondary infections [30–32], our work emphasize that B cells are also relevant for primary acute *Salmonella* infection. Taken together our data demonstrate that BAFF from Nphs and cDCs shapes early mature B cell responses and strengthens protective host responses against primary *S*. *typhimurium* infection. An improved understanding of niches of BAFF producing cells regulating B cell responses against *Salmonella*, as well as other harmful bacteria, may help designing novel strategies for vaccine and therapeutics development.

## Materials and methods

### Mice

C57BL/6J (B6), mice were purchased from Jackson Labs (Bar Harbor, ME) and housed or bred in our facilities at the University of Washington. BAFF-RFP and *Baff*^*fl/fl*^ mice (on the B6 background) were developed at the University of California at Davis Mouse Biology Program (UC Davis, CA). Generation of these mice strains are fully described elsewhere [9]. Briefly, BAFF-RFP reporter mice were generated by replacing a *Tnfsf13b/Baff* allele with a targeting construct expressing IRES-RFP. The *Tnfsf13b/Baff* is functionally knocked-out where the endogenous reporter expresses the RFP protein translated under the control on an IRES site. Thus, the BAFF-RFP signal is a measurement of BAFF expression. These heterozygous BAFF-RFP^+/-^ mice express the RFP protein on one *Baff* allele and the WT *Baff* on the other [9]. *Baff* ^*fl/fl*^ mice were used as controls as these mice express wild type *Baff* until Cre-mediated deletion. *Baff*^*fl/fl*^ mice were crossed with *Mrp8*^*Cre*^ mice [73], *zDC*^*Cre*^ mice [74] and *Cx3cr1*^*Cre*^ mice [73] to generate conditional knockout (cKO) mice where *Baff* is selectively deleted in either Nphs (*Baff*^*fl/fl*^ *Mrp8*^*Cre*^), cDCs (*Baff*^*fl/fl*^ *zDC*^*Cre*^) and MOs (*Baff*^*fl/fl*^ *Cx3cr1*^*Cre*^), respectively. All mouse strains were verified by sorting of appropriate cell subsets and assessing relative expression of *Baff* mRNA in sorted cells by qPCR [9].

Animals were housed under standard barrier conditions in groups of up to five animals in individually ventilated cages within a specific pathogen free environment. Mice were age- and sex-matched and used at 8–14 weeks of age.

### Ethics statement

This study was carried out in strict accordance with the recommendations in the Guide for the Care and Use of Laboratory Animals of the National Institutes of Health. All procedures were approved and conducted according to regulations of the University of Washington Institutional Animal Care and Use Committee (2242–08 and 4391–01). All research staff completed the required training for animal care or handling. Humane end points were defined prior to the experiments and all possible measures were taken to minimize animal suffering.

### Bacterial strains and mouse infection

The experiments were performed using *Salmonella enterica serovar Typhimurium* strain SL1344 kindly provided by Kelly Smith (University of Washington, Seattle, WA). The inoculum containing *S*. *typhimurium* was prepared as described [75]. Briefly, a single bacterial colony was grown overnight in Luria-Bertani (LB) broth containing 50 μg/ml of streptomycin at 37°C with shaking and back-diluted 1:50 in fresh media. After 4 hours, the OD (600 nm) of bacteria was measured and bacteria appropriately diluted in cold PBS. Mice were infected i.p. in a total volume of 200 μl with 7 or 25 CFU live bacteria for survival studies and with 500 CFU live bacteria for all other experiments. CFUs of the inoculi were verified by plating on LB agar plates containing 50 μg/ml streptomycin (LB/strep plates). Mice were monitored daily for 10 days or twice a day when clinical symptoms were expected to worsen. Survival, clinical scores and mice weights were recorded daily. Mouse survival is given as percentage of live animals per time point. Clinical scores and humane end points in response to systemic *S*. *typhimurium* infection were defined as described previously [76]. Clinical evaluations of mice were performed based on mice activity, feeding, appearance of fur, hunched position, ataxia and tremor. Humane endpoints were determined when mice showed severe clinical symptoms due to *S*. *typhimurium* infection and were immediately euthanized. Some mice during survival studies succumbed to infection before meeting criteria for euthanasia.

### Tissue harvest and cell isolations

Mice were sacrificed by CO_2_ asphyxiation at the indicated time points, and spleen and bone marrow (BM) were harvested for cell subset analyses. Mice and spleens were weighed, and weights were used for determining splenomegaly (mean of spleen weight/body weight (%)). A small part of each spleen was homogenized by 1.4 mm-bead disruption in a FastPrep-24 instrument (MP Biomedicals) in PBS with 0.025% Triton X-100. Bacterial burdens were assessed by plating serial dilutions of the spleen homogenates on LB/strep plates. Blood bacterial levels were assessed by immediately plating 100 ul of blood from retro-orbitol bleeds or serial dilutions of blood on LB/strep plates before the blood clotted. Preparation of splenic cell suspensions and isolation of BM cells and peritoneal exudate cells (PEC) from uninfected or *S*. *typhimurium*-infected mice were performed as described previously [9, 10, 77].

### Flow cytometry analysis

For flow cytometry analyses, single cell suspensions of splenocytes, BM cells or peritoneal exudate cells were counted and incubated at 4°C in PBS with an Aqua Live-Dead fixable viability dye (Molecular Probes, Life Technologies, Waltham, MA) to exclude the dead cells. Subsequently, cells were incubated with anti-Fc receptor Ab (anti-CD16/CD32) (2.4G2) (BioLegend, San Diego, CA) for 15 min at 4°C and then stained with various mAb mixtures for 20–30 min at 4°C. Cells were fixed in 2% paraformaldehyde and samples were acquired on an LSR II flow cytometer (Becton Dickinson, Franklin Lakes, NJ, USA) using a FACSDiva software; data were analyzed using FlowJo (v.10, Tree Star). The mAbs used in the study were conjugated to FITC, allophycocyanin, eFluor450, allophycocyanin-eFluor780, PerCPCy5.5, PE-Cy7, Alexa-Fluor647, BUV395, BV605, BV421, BV711, BV650, and BUV395. Eleven to twelve color flow cytometry was performed for cell subset analysis using combinations of the following mAbs: CD19 (1D3), CD11b (M1/70), CD11c (N418) and anti-CD69 (H1.2F3) from eBioscience; B220 (RA3-6B2), CD93 (AA4.1) and Ly6C (AL-21) from BD Horizon/Biosciences (San Jose, CA); and CD19 (1D3), B220 (RA3-6B2), CD3 (17A2), NK1.1 (PK136), CD8a (53–6.7), Ly6G (1A8), SiglecH (440c), Ly49c (14B11), CD49b (DX5), NKp46/CD335 (29A1.4), CD127 (SB/199), CD21/35 (7E9), CD23 (B3B4), CD24 (M1/69) and anti-MHC II (34-5-3) from BioLegend [9, 10, 77]. The BAFF-RFP signal was detected in the PE channel. Gating of splenic and BM myeloid cells were defined as described previously [9, 77]. For splenic myeloid cells, the B cells (CD19^+^CD3^-^) and T cells (CD19^-^CD3^+^) were gated out; non-B cells and non-T cells (CD19^-^ CD3^-^ gate) populations were defined as follows: Nphs, CD11b^hi^Ly6G^hi^Ly6C^int^SSC^int-^NK1.1^-^; Ly6Chi MOs, CD11b^hi^Ly6C^hi^CD11c^-^SSC^-^Ly6G^-^NK1.1^-^; Ly6C^hi^ DCs, CD11b^hi^Ly6C^hi^CD11c^hi^ SSC^-^Ly6G^-^NK1.1^-^; CD8^+^ cDCs, CD11c^hi^CD8^+^B220^-^Ly6G^-^NK1.1^-^; CD8^-^ cDCs, CD11c^hi^CD8^-^B220^-^Ly6G^-^NK1.1^-^. In BM, myeloid cells were defined in B220^-^CD19^-^NK1.1^-^SiglecH^-^CD11b^+^ gate as follows: Nphs, Ly6G^hi^Ly6C^int^SSC^int-^; Ly6Chi MOs, CD11c^-^CD115^+^CX3CR1^+^CCR2^hi^ MHCII^-^Ly6G^-^SSC^-^; preDCs, CD11c^+^Ly6C^-^CD115^+^CX3CR1^hi^CCR2^+^MHCII^+^Ly6G^-^SSC^-^. Splenic B cell subsets in the CD19^+^B220^+^ gate were defined as described previously [43] and are shown in S3A Fig in S1 File: follicular (FO) B cells, CD24^mid^CD21/35^mid^CD93^-^CD23^-^; marginal zone (MZ) B cells, CD24^hi^CD21/35^hi^ CD93^-^CD23^-^; MZ B cell precursors, CD24^hi^CD21/35^hi^CD93^lo^CD23^+^; T2 B cells, CD24^hi^CD21/35^int/hi^ CD93^+^CD23^+^; and T1 B cells, CD24^hi^CD21/35^lo^CD93^+^CD23^-^. Plasma cells (PCs) were defined as B220^lo^CD138^+^. Nphs in PEC were defined as CD19^-^B220^-^CD11b^hi^Ly6G^hi^Ly6C^int^SSC^int-^.

### Isolation of Nphs and stimulations assays

BMs from Naïve WT and BAFF-RFP^+/-^ mice were harvested; Nphs were enriched from BM cell suspensions using a magnetic-activated cell-sorting (MACS) neutrophil isolation kit (BM >95% purity, Miltenyi Biotec). Enriched Nphs were counted and seeded in 48 or 24 well plates at 2 or 4 x 10^6^ cells/well respectively in RPMI 1640 supplemented with 10% FBS at 37°C in 5% CO_2_. Nphs were stimulated with *S*. *typhimurium* whole cell lysates (200 μg/ml), live *S*. *typhimurium* (1:2 and 1:10 MOI) or G-CSF+GM-CSF (100 ng/ml) for 6 or 24 hrs. Unstimulated Nphs were used as a negative control. The *S*. *typhimurium* whole cell lysate was prepared by homogenizing an overnight bacterial culture by 0.1 mm-bead disruption in a FastPrep-24 instrument (MP Biomedicals) in PBS. The protein content of the lysate was measured using Pierce BCA protein assay kit as per manufactures instructions. After incubation the cell supernatants were collected and stored at -80°C. Half of the Nph cells were processed for flow cytometry to assess the BAFF-RFP signal; the other half of the cells were lysed and RNA extracted for BAFF qRT-PCR analyses as described previously [9].

### *Salmonella* specific Ab and BAFF protein detection

Sera were isolated from blood, collected via the retro-orbital route, and stored at -80°C until use. *Salmonella***-**specific serum IgM Ab were detected by ELISA. Maxisorp plates (Nunc) were coated with 50 μg/ml *Salmonella* whole cell lysate and the amount of specific Ab were determined as described previously using a standard curve [77]. Briefly, 2 μg/ml anti-mouse IgM (Jackson ImmunoResearch), were coated, and serial dilutions of known concentration of recombinant IgM (Southern Biotech, Birmingham, AL) were used for the standard curve. Serum samples and recombinant IgM were diluted in 4% milk casein in PBS containing 0.05% Tween-20, and primary mouse Abs were detected using isotype specific goat anti-mouse antibodies conjugated to horseradish peroxidase (Southern Biotech, Birmingham, AL). Serum levels of BAFF from *in vivo* experiments and in supernatants from stimulated Nph cultures were determined using the Mouse BAFF/BLyS/TNFSF13B DuoSet ELISA kit (R&D systems, Inc., Minneapolis, MN) according to the manufacturer’s instructions.

### Statistical analyses

All statistical analyses were performed with Prism software (GraphPad Software). A *p* value of < 0.05 was considered significant. Survival data were analyzed by Mantel-Cox log-rank test. Statistical analyses of flow cytometry experiments and BAFF protein detected by ELISA comparing more than two groups were performed with One-Way ANOVA corrected for multiple comparisons using the Holm-Sidak method. Analyses between two groups were performed using unpaired Student *t* test. Flow cytometry and BAFF protein experiments involving BAFF cKO mice, timeline data of clinical scores, mouse weights, and IgM Ab assays of various groups were analyzed using a Two-way ANOVA with Holm-Sidak’s multiple comparison test.

## Supporting information

S1 File (PDF)
