# Supplementary material for: B cell activating factor (BAFF) from neutrophils and dendritic cells is required for protective B cell responses against Salmonella typhimurium infection
Source: PLoS One. 2021 Oct 27;16(10):e0259158. doi: 10.1371/journal.pone.0259158 (PMC8550399; doi:10.1371/journal.pone.0259158)
Supplement: S1 File — (PDF) [file pone.0259158.s001.pdf]

Supplemental material to Kuley et al 2021:

B cell activating factor (BAFF) from neutrophils and dendritic cells is required for protective B cell responses against *Salmonella typhimurium* infection

Runa Kuley[1,2]*, Kevin E. Draves[2,3], Deborah H. Fuller[3], Natalia V. Giltiay[1], Edward A. Clark[1,2,3] and Daniela Giordano[1,2]*

[1]Department of Medicine, Division of Rheumatology, University of Washington, Seattle, WA, USA.

[2] Department of Immunology, University of Washington, Seattle, WA, USA

[3] Department of Microbiology, University of Washington, Seattle, WA, USA

Corresponding authors:

Email: kuleyr@uw.edu (RK)

giordano@uw.edu (DG; Co-corresponding author)

S1 Fig.

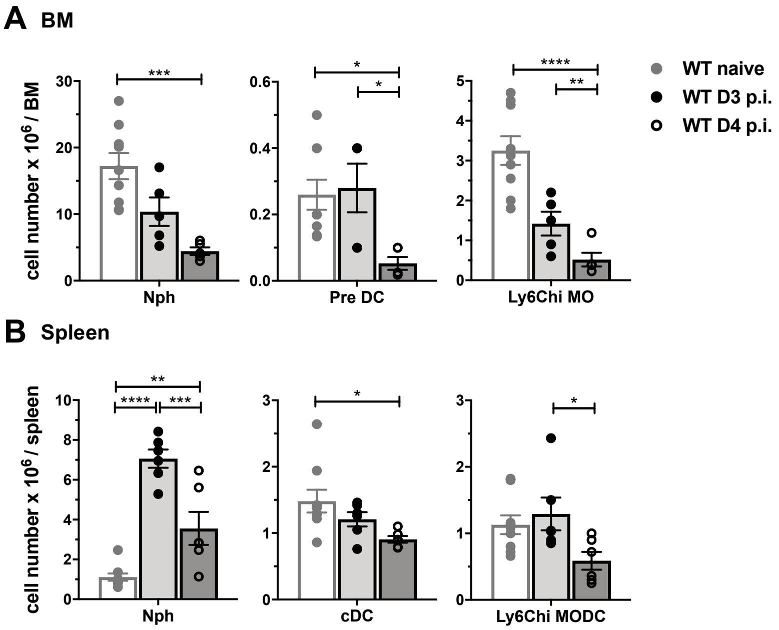

S1 Fig. Cellular responses in WT mice after *S. typhimurium* infection. Cells from BMs and spleens were harvested at day 3 and 4 p.i., and myeloid cell subsets were determined by flow cytometry. (A) Numbers of BM Nphs ($CD11b^{+}Ly6G^{hi}Ly6C^{int}SSC^{int-}$), Pre DCs ($CD11b^{+}CD11c^{+}Ly6C^{-}CD115^{+}CX3CR1^{hi}CCR2^{+}$ $MHCII^{+}Ly6G^{-}SSC^{-}$) and $Ly6C^{hi}$ MOs ($CD11b^{+}CD11c^{-}CD115^{+}CX3CR1^{+}CCR2^{hi}$ $MHCII^{-}Ly6G^{-}SSC^{-}$) from BAFF-$RFP^{+/-}$ mice after *S. typhimurium* infection. Graphs summarize data from three independent experiments (n = 6-9). (B) Numbers of splenic Nphs ($CD11b^{hi}Ly6G^{hi}Ly6C^{int}$ $SSC^{int-}NK1.1^{-}$), cDCs ($CD11c^{hi}CD8^{+/-}$ $B220^{-}Ly6G^{-}NK1.1^{-}$) and $Ly6C^{hi}$ MO/DCs ($CD11b^{hi}$ $Ly6C^{hi}CD11c^{hi/-}SSC^{-}Ly6G^{-}NK1.1^{-}$) from BAFF-$RFP^{+/-}$ mice after *S. typhimurium* infection. Data are combined from four independent experiments (n = 6-9). Bar graphs show means ± SEM; * p <0.05, ** p <0.01, *** p <0.001, **** p <0.0001, as determined by One-Way Anova with Holm-Sidak multiple comparisons test.

S2 Fig.

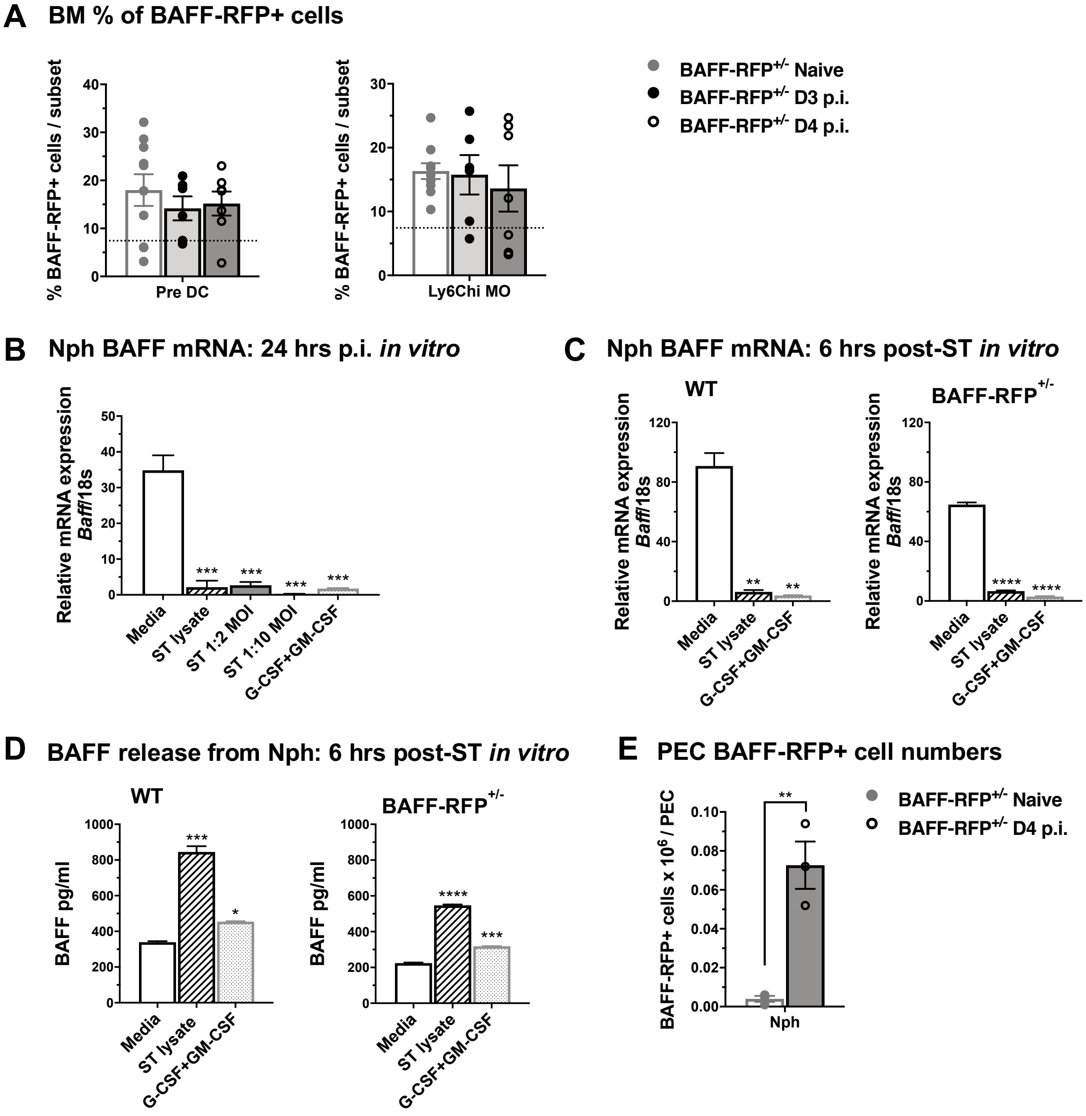

S2 Fig. BAFF regulation in BM myeloid cells and Nphs after *S. typhimurium* infection. (**A** and **E**) BAFF-RFP$^{+/-}$ mice were infected i.p. with 500 CFU *S. typhimurium*. BM (**A**) and peritoneal exudate cells (PEC) (**E**) were harvested and analyzed by flow cytometry. (**A**) Bar graphs (means ± SEM) indicate the percentage of BAFF-RFP^{+} preDC and MO cells. Dotted lines show the percentage of RFP background signal in WT cells. (**A**) Data are combined from three independent experiments (n = 6-9). (**B-D**) BM Nphs were isolated from WT and BAFF-RFP$^{+/-}$

mice and stimulated for 6 or 24 hrs with live *S. typhimurium* or bacterial lysates *in vitro*. (**B** and **C**) *Baff* mRNA expression analyzed by quantitative PCR and shown as arbitrary units relative to 18S. (**B**) Bar graphs show data from purified WT Nphs stimulated with *S. typhimurium* for 24 hrs. (**C**) Bar graphs show data from WT Nphs and BAFF-RFP$^{+/-}$ Nphs treated for 6 hrs with bacterial lysates. (**D**) BAFF titers were analyzed by ELISA from supernatants of WT Nphs and BAFF-RFP$^{+/-}$ Nphs stimulated for 6 hrs with live *S. typhimurium* or bacterial lysates. (**B**-**D**) G-CSF plus GM-CSF were used as positive controls. (**B**-**D**) Data show one representative of two independent experiments. (**E**) Bar graphs (means ± SEM) indicate the numbers of BAFF-RFP^{+} Nphs (CD11b^{hi}Ly6G^{hi}Ly6C^{int}SSC^{int-}) are shown at day 4 p.i. from peritoneal exudate cells (PEC). Data are from one experiment with 3 mice per group. (**A-D**) Statistics were determined by One-Way Anova with Holm-Sidak multiple comparisons test and (**E**) unpaired Student *t* test.; * p <0.05, ** p <0.01, *** p <0.001, **** p <0.0001. (ST refers to *S. typhimurium*).

S3 Fig.

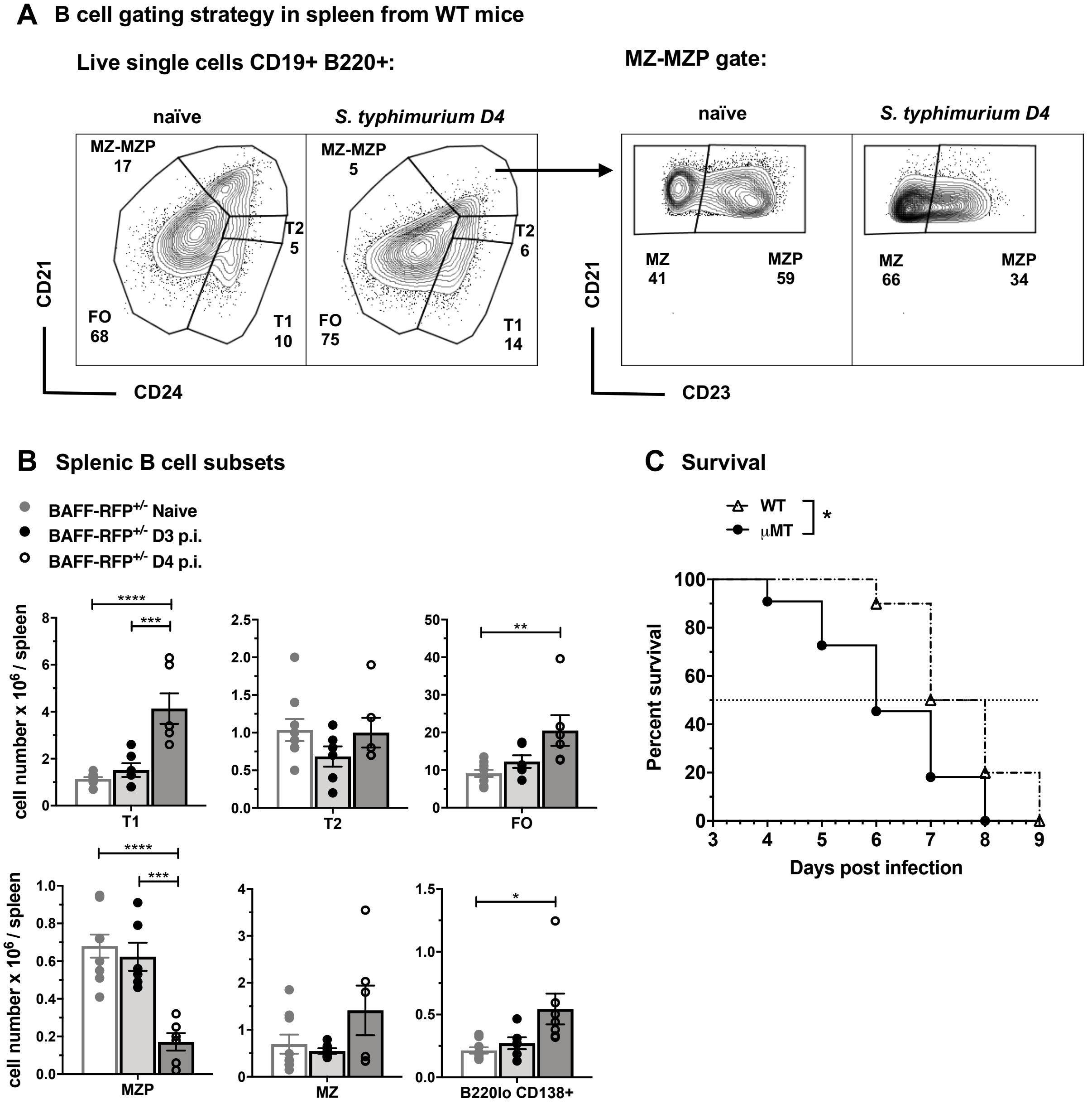

S3 Fig. *S. typhimurium* induces expansion of T1, FO B cells and plasma cells in WT and BAFF-RFP$^{+/-}$ mice and susceptibility of μMT mice to infection with *S. typhimurium.* (**A** and **B**) WT and BAFF-RFP$^{+/-}$ mice were infected i.p. with 500 CFU *S. typhimurium*, spleens were harvested, and B cell subsets were assessed by flow cytometry. (**A**), Gating strategy of splenic B cell subsets in naïve and infected WT mice: T1 B cells, CD24^{hi}CD21/ 35^{lo}CD93^{+}CD23^{-}; T2 B

cells, $CD24^{hi}CD21/35^{int/hi}CD93^{+}CD23^{+}$; FO B cells, $CD24^{mid}CD21/35^{mid}CD93^{-}CD23^{-}$; MZ, marginal zone B cells, $CD24^{hi}CD21/35^{hi}$ $CD93^{-}CD23^{-}$; MZP, MZ precursors, $CD24^{hi}CD21/35^{hi}CD93^{lo}CD23^{+}$ and plasma cells, B220lo $CD138^{+}$. T1, T2 and MZ-MZP gates were assessed overlaying CD93 and CD23 on the CD21 vs CD24 dot plots using the heatmap. The numbers in the dot plots indicate % of cells in each subset, and the data are representative of one out of three independent experiments. (**B)**, Absolute numbers of splenic B cell subsets in naïve and infected BAFF-$RFP^{+/-}$ mice. Graphs summarize data from three independent experiments (n = 6-9). Bar graphs show means ± SEM; * p <0.05, ** p <0.01, *** p <0.001, **** p <0.0001, as determined by One-Way Anova with Holm-Sidak multiple comparisons test. (**C)**, µMT (B cell deficient) mice were infected i.p. with 7 CFU *S. typhimurium*. Mice were monitored daily for survival and survival data are indicated as a percentage of live animals per time point. Data are combined from two independent experiments: WT, n = 10; and uMT, n = 11. Statistics were performed using a log-rank test for significance *p <0.05.

S4 Fig.

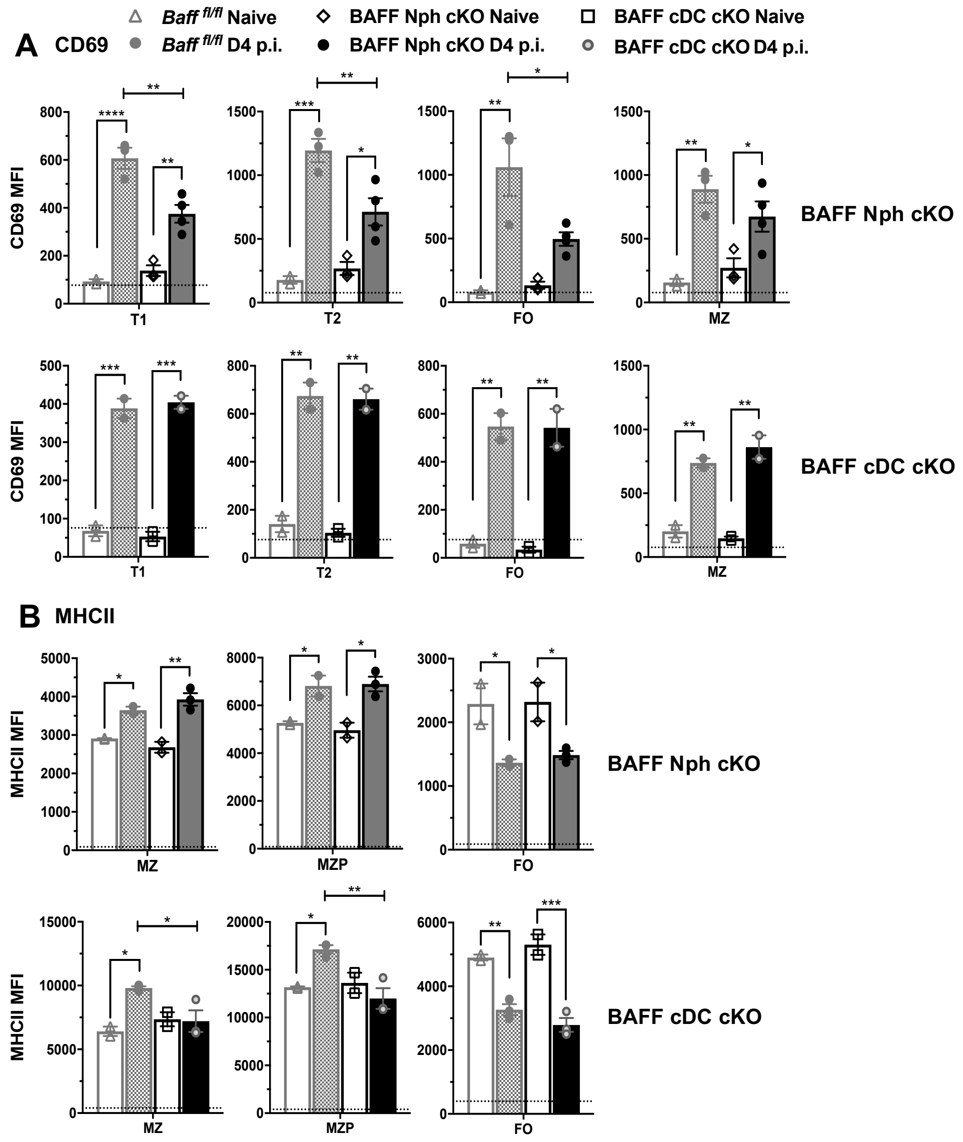

S4 Fig. BAFF from Nph and cDCs have differential effects on B cell activation. *Baff$^{fl/fl}$*, *Baff$^{fl/fl}$ MRP8^{Cre}* (BAFF Nph cKO) and *Baff$^{fl/fl}$ zDC^{Cre}* (BAFF cDC cKO) mice were infected i.p. with 500 CFU *S. typhimurium.* Spleens were harvested from naïve and day 4 infected mice and activation markers in B cell subsets were analyzed by flow cytometry. Graphs show CD69 MFI (**A**) and MHCII MFI (**B**). (**A** and **B**) Data show one representative of two independent experiments (n = 2-4). Bar graphs show means ± SEM and dotted line indicate background from

unstained controls. Statistics were performed using Two-Way Anova with Holm-Sidak's multiple comparisons test; * $p < 0.05$, ** $p < 0.01$, *** $p < 0.001$, **** $p < 0.0001$.

S5 Fig.

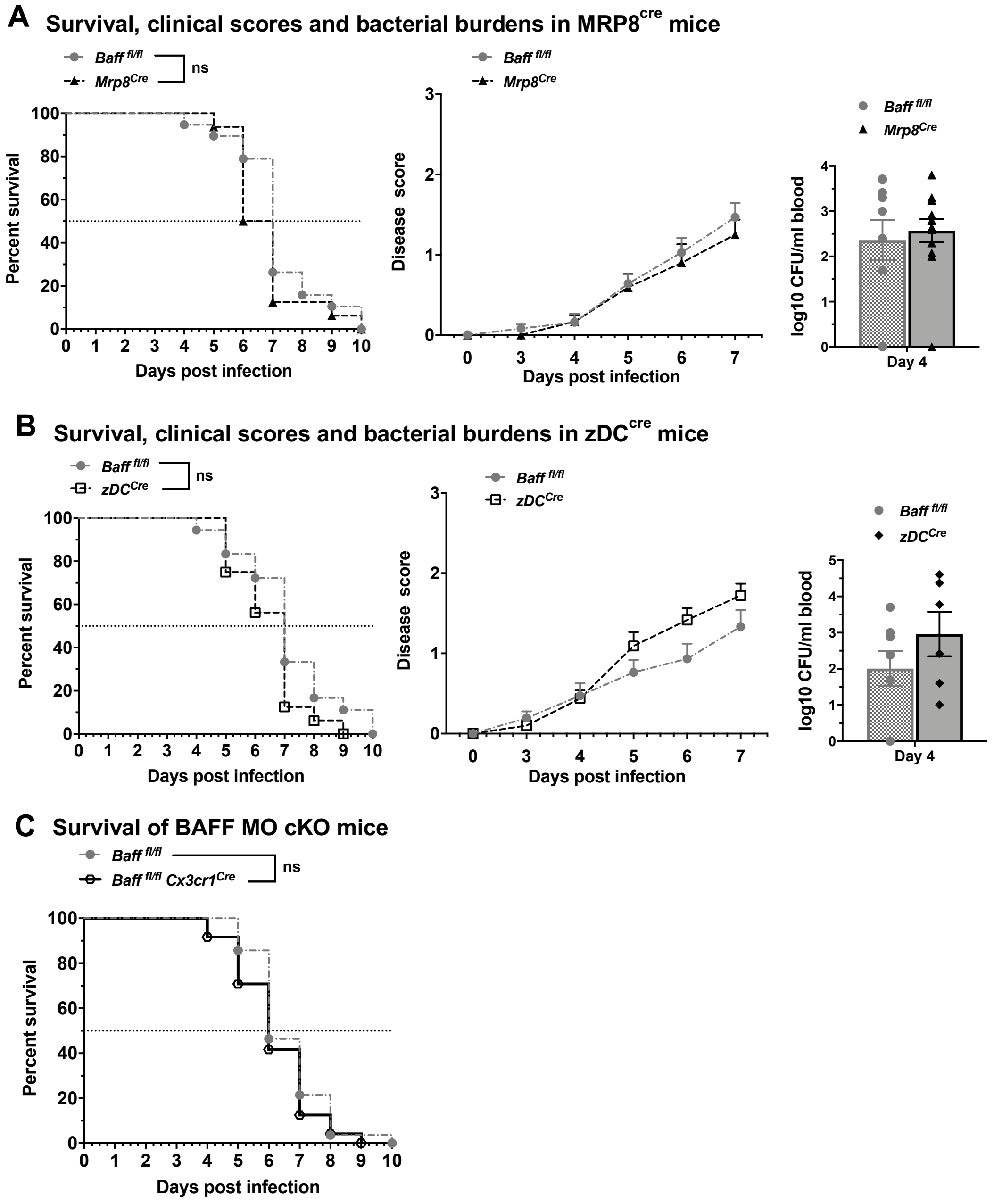

S5 Fig: *MRP8*^{Cre} mice, *zDC*^{Cre} mice and *Baff*$^{fl/fl}$ *Cx3cr1*^{Cre} mice have similar survival rates as control *Baff*$^{fl/fl}$ mice. *Baff*$^{fl/fl}$, *MRP8*^{Cre}, *zDC*^{Cre} and *Baff*$^{fl/fl}$ *Cx3cr1*^{Cre} (BAFF MO cKO) mice were infected i.p. with 25 CFU *S. typhimurium.* (**A and B**) Data show survival (*left panel*), clinical scores (*middle panel*) and bacterial burdens measured at day 4 p.i. (*right panel*). (**A and B**) Survival and clinical scores data are combined from three independent experiments: *Baff*$^{fl/fl}$,

n=19; *MRP8*^{Cre}, n=16 and *zDC*^{Cre}, n=17. Data of bacterial burdens were summarized from two independent experiments (n = 6-13). (**C**) Survival data are combined from three independent experiments: *Baff*$^{fl/fl}$, n=24; BAFF MO cKO, n=24. Survival data were analyzed using a log-rank test for significance and significance of clinical scores were determined by Two-Way Anova with Holm-Sidak's multiple comparisons test; $p > 0.05$ (not-significant). The unpaired Student *t* test was used to determine significance in bacterial burdens. Bar graphs show means ± SEM; ns=not-significant ($p > 0.05$).
